# Genome-Wide Identification of the DnaJ Gene Family in Citrus and Functional Characterization of *ClDJC24* in Response to Citrus Huanglongbing

**DOI:** 10.3390/ijms252211967

**Published:** 2024-11-07

**Authors:** Yuzhen Tian, Xizi Wang, Huoqing Huang, Xin Deng, Baihong Zhang, Yixuan Meng, Libo Wu, Hang Chen, Yun Zhong, Wenli Chen

**Affiliations:** 1 MOE Key Laboratory of Laser Life Science & Institute of Laser Life Science, Guangdong Provincial Key Laboratory of Laser Life Science, Guangzhou Key Laboratory of Spectral Analysis and Functional Probes, College of Biophotonics, School of Optoelectronic Science and Engineering, South China Normal University, Guangzhou 510631, China; tianyzh@126.com (Y.T.); wangxizi1103@163.com (X.W.); 2021023392@m.scnu.com (Y.M.); 2023023373@m.scnu.edu.cn (L.W.); ccc2020xxsc@126.com (H.C.); 2 Institute of Fruit Tree Research, Guangdong Academy of Agricultural Sciences, Key Laboratory of South Sub-Tropical Fruit Biology and Genetic Research Utilization, Ministry of Agriculture and Rural Affairs, Guangdong Provincial Key Laboratory of Science and Technology Research on Fruit Tree, Guangzhou 510640, China; hqhuang07@163.com; 3 Department of Biomedical Sciences, City University of Hong Kong, Kowloon Tong, Hong Kong SAR, China; xindeng@cityu.edu.hk; 4 Shenzhen Research Institute, City University of Hong Kong, Shenzhen 518057, China; 5 Institute of Nanfan & Seed Industry, Guangdong Academy of Science, Guangzhou 510640, China; zhangbyhome@126.com

**Keywords:** *DnaJ*, citrus, huanglongbing (HLB), disease resistance

## Abstract

Citrus Huanglongbing (HLB) is the most destructive citrus disease worldwide. The etiological agent responsible for this disease is “*Candidatus* Liberibacter asiaticus” (*C*Las), a phloem-restricted bacterium transmitted by psyllid vectors. To date, effective practical strategies for curing Citrus HLB remain elusive. Additionally, no susceptibility genes associated with HLB have been identified in Citrus species, thereby complicating the application of gene-editing techniques such as CRISPR-Cas9 to enhance resistance to HLB. The co-chaperone *DnaJ* plays a crucial role in protein folding and the regulation of various physiological activities, and it is also associated with multiple pathological processes. *DnaJ* has been extensively studied in many species, including Arabidopsis, rice, and wheat. However, there is limited information available regarding the DnaJ gene family in citrus. In this study, we conducted a comprehensive genome-wide analysis of the DnaJ family genes in various Citrus species. The Citrus genome was identified to contain 86 *DnaJ* genes, which were unevenly distributed across nine chromosomes. Phylogenetic analysis indicated that these genes could be classified into six distinct groups. Furthermore, transcriptomic analysis revealed that nine *DnaJ* genes exhibited significantly higher induction in HLB-infected samples relative to non-HLB-infected Citrus. *Cis*-acting elements within the promoters of *DnaJ* genes were also examined, revealing the presence of hormone and defense/stress responsiveness elements (TC-rich) distributed on the *ClDJC24* gene. The results were validated using quantitative real-time PCR (qRT-PCR). Additionally, the silencing of *ClDJC24* suggested that this gene negatively regulates disease resistance in Citrus. Our study provided useful clues for further functional characterization and constructed a theoretical foundation for disease-resistant breeding in Citrus.

## 1. Introduction

Heat shock proteins (HSPs), the most extensive class of molecular chaperones, safeguard the interacting surfaces of protein substrates from conformational damage. They achieve this by binding to the substrates and promoting the proper folding of unfolded or nascent polypeptides [1]. HSPs can be categorized into six distinct families based on their molecular weights: HSP100, HSP90, HSP70, HSP60, HSP40, and small heat shock proteins [2,3]. DnaJ is a prototypical member of the HSP40 family, characterized by an N-terminal conserved domain known as the J domain, a glycine- and phenylalanine-rich (G/F-rich) domain, a central cysteine-rich domain (CR-type zinc finger) that includes four repeats of a CxxCxGxG motif (zinc finger-like region), and a substrate-binding C-terminal domain [4,5,6]. The J domain binds to HSP70 and stimulates the ATPase activity of co-chaperone proteins to facilitate protein folding, unfolding, translocation, and degradation [7]. For example, DnaJ proteins have been documented to facilitate the regulation of growth, development, and environmental adaptation in both prokaryotic and eukaryotic organisms, including fungi and plants [8,9,10]. The HSP40/DnaJ protein family is one of the most diverse families of auxiliary molecular chaperones [6]. *DnaJ*, also known as HSP40 or J-proteins, recognizes unfolded substrates and delivers them to DnaK. It facilitates ATP hydrolysis in HSP70s and induces conformational changes in stable chaperone proteins [11]. The DnaJ family represents the most diverse group of co-chaperones, distinguished by conserved J-domains initially identified in *Escherichia coli*. Members of the DnaJ family also contain DnaJ protein and a highly conserved HPD tripeptide signature motif [12]. HSP70 plays a crucial role in multiple stages of the viral lifecycle, making it one of the multifunctional proteins involved in regulating viral infection by the host [13]. Research indicates that HSP70 typically does not function in isolation; its activity relies on the assistance of HSP40/DnaJ proteins for regulation [6]. There may be multiple ways of interacting between DnaJ family proteins and HSP70 proteins, and regulating the function of HSP70 through different interaction ways may be the key to the wide range of cellular functions of HSP70 proteins [14].

Previous studies have identified 117 DnaJ homologs in Arabidopsis [15], 115 in rice [16], and 88 in wheat [12]. However, there is limited research about Citrus DnaJ. DnaJ proteins play diverse physiological roles, including hormone regulation and disease resistance [17,18,19]. The Arabidopsis AtJ1 protein is crucial for the abscisic acid (ABA) response [20], while the expression of the J-protein LeCDJ2 is induced by salicylic acid (SA) [21]. Moreover, the movement protein of the tobacco mosaic virus has been shown to interact with a DnaJ-like protein [22,23,24], and the interaction between DnaJ and soybean mosaic virus coat protein leads to susceptibility [25]. Additionally, various members of the DnaJ protein family play critical roles in regulating the replication and pathogenesis of diverse viruses [26].

Citrus Huanglongbing (HLB) is caused by the phloem-inhabiting, Gram-negative α-proteobacterium “*Candidatus* Liberibacter asiaticus” (*C*Las). This pathogen significantly reduces yield and leads to considerable economic losses in citrus production, ultimately jeopardizing the health of the entire tree [27,28]. Despite extensive scientific investigations, no cure for HLB has yet been discovered. To date, the DnaJ gene family has been extensively studied in rice, wheat, and Arabidopsis. DnaJ proteins have been identified in various species and are generally associated with plant growth, development, and stress resistance, among other functions [29]. In addition, research has shown that the DnaJ gene family is involved in the defense mechanisms of plants in wheat [12]. However, there is a paucity of information regarding the DnaJ gene family in Citrus.

In the present study, the DnaJ family members were identified throughout the Citrus genome. Gene structures, conserved domains, phylogenetic relationships, chromosomal locations, and expression patterns of all 86 *DnaJ* genes in Citrus were also analyzed. According to the results of *ClDJC24* expression analysis, the role of *ClDJC24* was explored further. Subsequently, the functions of the *DnaJ* genes were elucidated through the evaluation of gene silencing effects in Citrus plants during HLB infection. This study provided novel insights for resistant gene resources and provided useful clues for further functional characterization in Citrus.

## 2. Results

### 2.1. Genome-Wide Identification of DnaJ Genes from Citrus

According to the characteristics of the DnaJ domain (Pfam: PF00226), all 86 DnaJ protein sequences were identified from *Citrus clementina*. As illustrated in Table 1, the number of amino acids (AAs), molecular weight (Mw), and theoretical pI varied from 101 (Ciclev10026825m.1) to 2592 (Ciclev10007224m.1), 12.00 (Ciclev10010382m.1) to283.90 (Ciclev10007224m.1) kDa, and 4.64 (Ciclev10016883m.1) to 10.07 (Ciclev10003089m.1), respectively (Table 2). Additionally, the subcellular localization of all members was predicted using WoLF PSORT, and the results indicated that most members (such as Ciclev10029600m.1 (*ClDJC24*) are localized in the nucleus (nucl), with a few localized in the chloroplast (chlo) and cyto (Table 1).

### 2.2. Chromosomal Distribution of DnaJ Genes from Citrus

**Figure 1:**
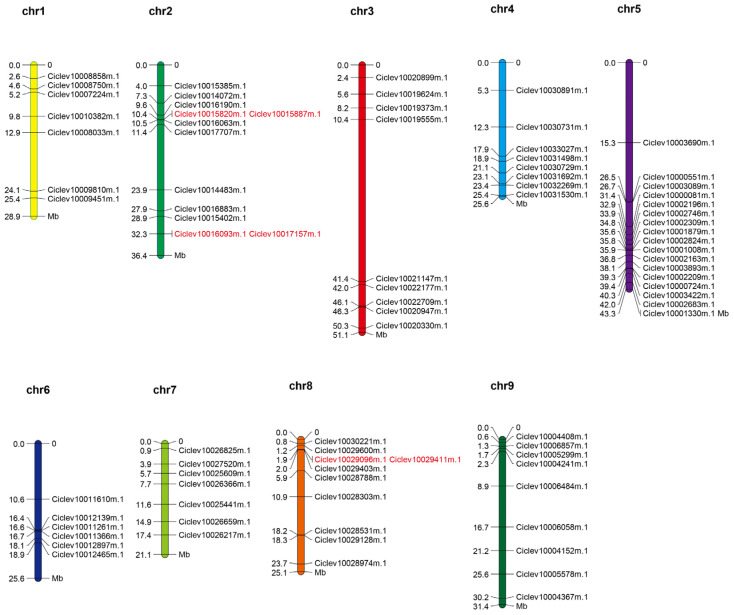
Chromosomal locations of *DnaJ* genes in Citrus. The chromosomal position of each *DnaJ* gene was mapped according to the genome of Citrus. The number at the top of each chromosome represents the chromosome number, and the scale unit is megabases (Mb). The gene duplication events are marked in red.

Chromosomal distribution and the tandem duplications of the *DnaJ* genes from Citrus were analyzed (Figure 1). As illustrated in Figure 1, analysis of the 86 *DnaJ* genes from citrus genome chromosomal locations showed that they were unevenly anchored to nine chromosomes (Chr) Chr1-Chr9 by MapChart1.0 software. Most *DnaJ* genes were from Chr5 (15 DnaJ genes) of citrus, followed by Chr2 (10 DnaJ genes). In contrast, six genes were found on Chr6. These results indicate that *DnaJ* genes from Citrus are distributed unevenly throughout the genome. Furthermore, according to the analysis, four genes were clustered into two tandem duplication events (Ciclev10015820m.1/Ciclev10015887m.1 and Ciclev10016093m.1/Ciclev10017157m.1) on Chr2 (Figure 1). And Chr8 contained a tandem duplication event of Ciclev10029096m.1 and Ciclev10029411m.1. These results suggest that some of the *DnaJ* genes from citrus might be produced by gene duplication, which undoubtedly promotes their evolution.

### 2.3. Gene Structures and Motif Analysis of DnaJ Genes from Citrus

**Figure 2:**
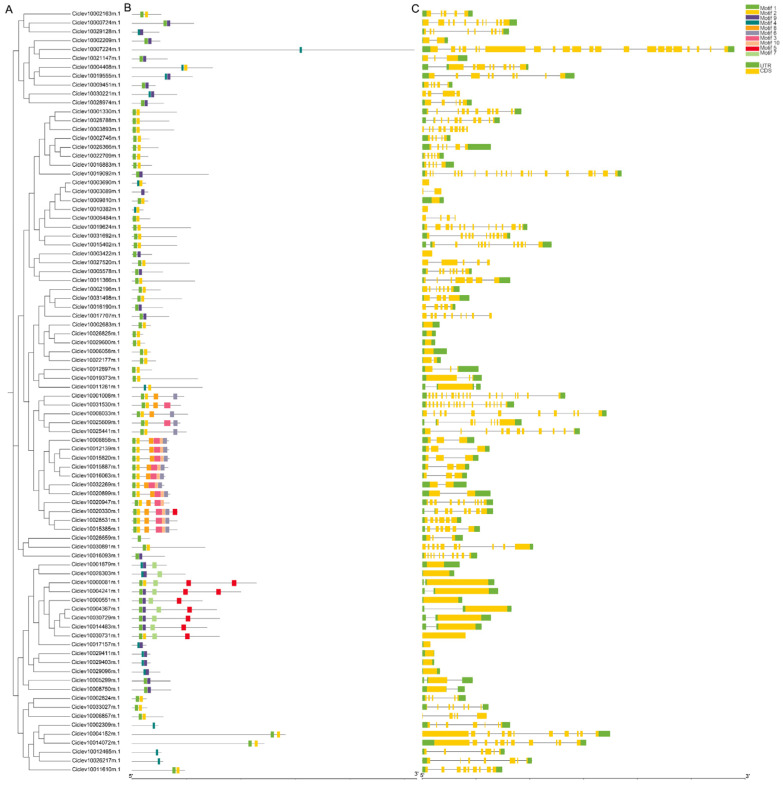
The gene structure analysis of *DnaJ* gene family from citrus. (**A**) Phylogenetic relationship of *DnaJ* genes from citrus. (**B**) Conserved motifs analysis of the DnaJ from Citrus proteins. All motifs were identified by the MEME database with the complete amino acid sequences of the *DnaJs*. The detailed information for each motif is provided in Figure S1. (**C**) Gene structures of Citrus DnaJ members. Yellow boxes and gray lines indicate exons and introns, respectively, and the UTR is marked in green.

A multiple sequence alignment was performed on the protein sequences derived from 86 *DnaJ* genes in citrus. Subsequently, a phylogenetic tree was constructed using the neighbor-joining (NJ) method in MEGA-X with 1000 bootstrap replicates (Figure 2A). As shown in Figure 2B,C, the conserved motifs and gene structures of 86 *DnaJ* genes were visualized by TBtools. The motif architectures motif 1 and motif 2 were the two most frequently presented motifs, covering almost all *DnaJ* gene families according to frequencies of occurrence (Figure 2B). Motif 9 was a specific motif that was solely found in Ciclev10003089m.1. Additionally, Motif 4 was a specific motif in Ciclev10002309m.1, Ciclev10012465m.1, Ciclev10007224m.1, and Ciclev10026217m.1. Ciclev10020330m.1 contained the largest number of motifs, including in motif 1, motif 2, motif 3, motif 6, motif 8, motif 9, and motif 10. To gain more insight into the evolutionary relationships within the Citrus *DnaJ* genes, we examined the exon and intron structures of all *DnaJ* gene members. In Figure 2C, among the 86 *DnaJ* genes, 13 had no introns, whereas the other *DnaJ* genes possessed 1 to 21 exons (9 with one exon, 12 with two exons, 2 with three exons, 7 with four exons, 6 with five exons, 9 with six exons, 2 with ten exons 2 with sixteen exons, 1 with twenty exons and 1 with twenty-one exons) (Figure 2C). These results indicated varying degrees of evolutionary conservation and divergence between the *DnaJ* genes from Citrus.

### 2.4. Characterization of Citrus HLB-Related DnaJ Genes

**Figure 3:**
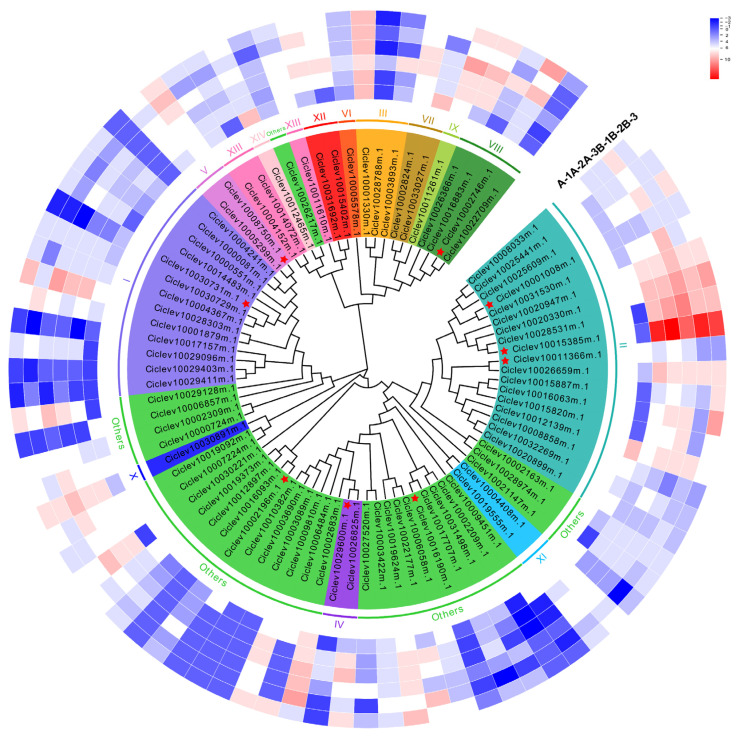
Phylogenetic analysis and expression patterns analysis of DnaJ proteins in Citrus with HLB. The clades are indicated by different frame colors. Amino acid sequences alignment of 86 DnaJ proteins in citrus. The heat map was generated using TBtools. The bar at the right of the heat map represents relative expression values. In the transcriptome data, sample A (A-1, A-2, A-3): post-treatment sample (new tissues), 90 days after FOS and CAC combination therapy; Sample B (B-1, B-2, B-3): pre-treatment sample. A-1, A-2, A-3, and B-1, B-2, B-3 represented no HLB-infected and HLB-infected with three biological replicates, respectively. Red star indicates the nine genes identified based on transcriptome data.

To investigate the evolutionary characteristics of the DnaJ genes family, we conducted the phylogenetic and clades analyses of DnaJ protein sequences from Arabidopsis and Citrus according to the classification criteria of Zhang et al. in Arabidopsis and Brassica [15]. Figure S2 shows that the phylogenetic tree divided 86 DnaJ proteins into 15 major clades (I-XV). The gene names of the 86 DnaJ proteins in Citrus, Arabidopsis ortholog, and the clades analysis are presented in Table 1. As shown in Figure 3, the clades are indicated by different frame colors. The study by Zhang et al. indicates that J-proteins in *Arabidopsis thaliana* and *brassica oleracea* are grouped into 15 major clades (I–XV), often separated by low-confidence branches that may represent unrelated single-gene clades. Clades I and II, each containing more than 10 members in Arabidopsis and B. oleracea, are classified as multigene clades. Clades III–XV contain 2–7 members and are termed oligogene clades. The remaining clades, with a single member in at least one of the two species, are designated as monogene clades [15]. In Figure 3, the largest number of *DnaJ* genes was observed in clade II with 18 *DnaJ* genes, followed by subgroup I (13) and clade VIII (4). However, clades VI, IX, X, XIII, and XIV only contained 1 DnaJ member from Citrus. In this study, candidate gene Ciclev10029600m.1 (*ClDJC24*) was notably divided into clade IV. The above results indicated the distinct phylogenetic characteristics of *DnaJ* genes in Citrus. Functional analysis of these genes might provide more information for the evolution of the DnaJ gene family.

To identify *DnaJ* genes related to citrus HLB, transcriptome data from control and HLB-infected citrus were obtained from earlier experiments generated in this lab [30]. The heatmap was created based on fragments per kilobase of transcript per million fragments mapped (FPKM) values from no HLB-infected and HLB-infected samples (Table S3). In the transcriptome data, sample A (A-1, A-2, A-3): post-treatment sample (new tissues), 90 days after FOS and CAC combination therapy; Sample B (B-1, B-2, B-3): pre-treatment sample. A-1, A-2, A-3, and B-1, B-2, B-3 represented no HLB-infected and HLB-infected with three biological replicates in this study, respectively. As shown in Figure 3, based on transcriptome data, nine genes were screened and indicated with asterisks.

### 2.5. Cis-Acting Elements in DnaJ Genes Promoters from Citrus

**Figure 4:**
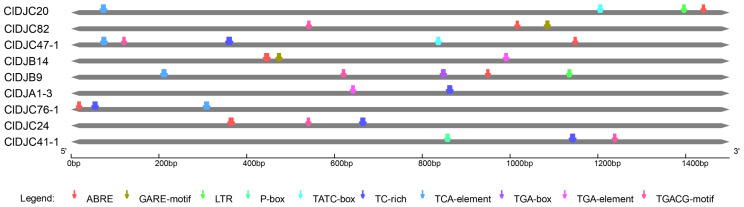
Cis-element analysis of 9 selected *DnaJ* genes from the upstream 1500 bp sequence to the transcription start site. Note: auxin responsive element (TGA-element, AACGAC; TGA-box, TGACGTAA), defense and stress responsiveness (TC-rich repeats, GTTTTCTTAC), gibberellin responsive element (GARE-motif, TCTGTTG; P-box, CCTTTTG; TATC-box, TATCCCA), low-temperature responsiveness (LTR, CCGAAA), MeJA responsiveness (TGACG-motif, TGACG; CGTCA), salicylic acid responsiveness (TCA-element, CCATCTTTTT), abscisic acid responsiveness (ABRE, ACGTG).

To understand the functional properties of *DnaJ* gene family members, we predicted cis-acting elements in the promoter regions using the PlantCARE database. In Figure 4, cis-elements of the nine *DnaJ* genes are illustrated to further examine the regulatory mechanisms of *DnaJ* genes related to Citrus HLB. The findings disclosed a variety of *cis*-acting elements, encompassing those associated with defense and stress responsiveness, low-temperature responsiveness, drought inducibility, and hormone responsiveness. As illustrated in Figure 4, defense and stress response (TC-rich, GTTTTCTTAC) were discovered in five genes, whereas they were not detected in the other three *DnaJ* genes. In addition, four contained TCA-element (CCATCTTTTT), which is a salicylic acid (SA) responsive element (Figure 4). Ciclev10029600m.1 (*ClDJC24*) included MeJA responsiveness elements (TGACG-motif, TGACG and CGTCA), abscisic acid responsiveness (ABRE, ACGTG), and defense and stress responsiveness elements (TC-rich repeats, GTTTTCTTAC) (Figure 4). These results suggest that *DnaJ* genes play important roles in Citrus defense and stress and hormone responses.

### 2.6. Relative Expressions of Citrus HLB-Related 9 DnaJ Genes

**Figure 5:**
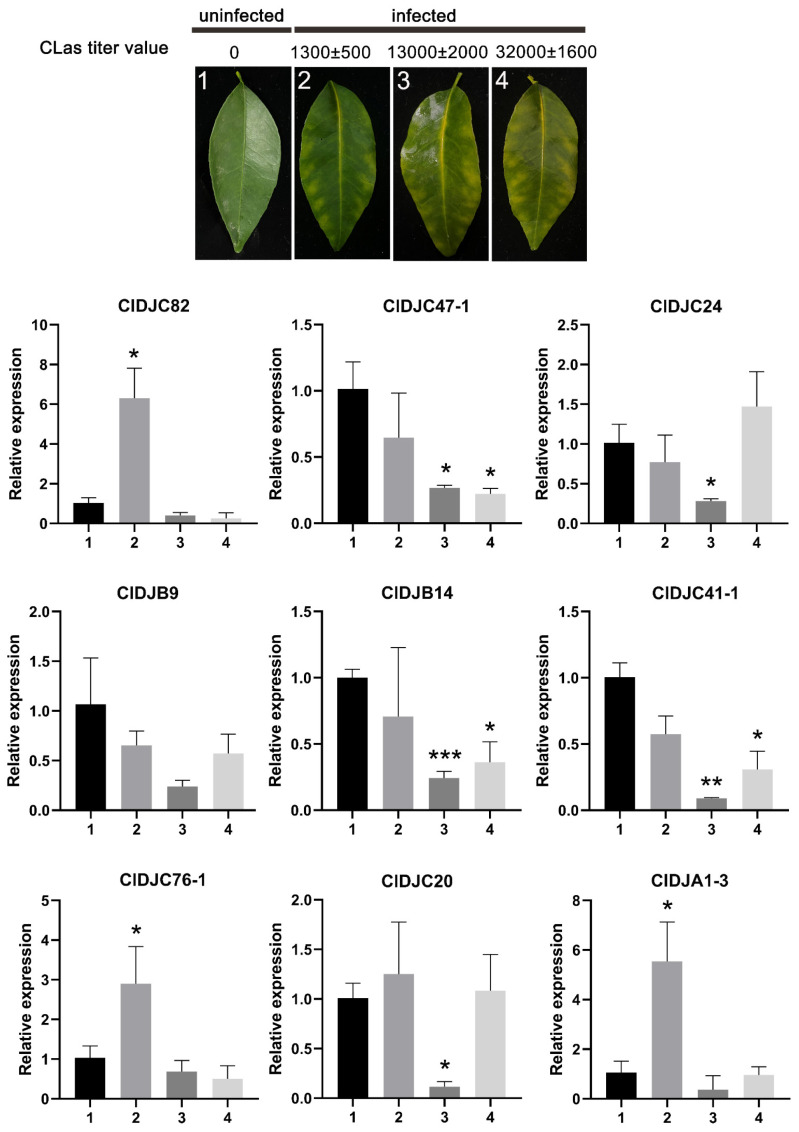
Relative expression of 9 selected *DnaJ* genes in groups 1, 2, 3, and 4. *C*Las titer refers to the bacterial load in 100 ng of genomic DNA. Based on the symptoms and *C*Las content, the leaves were divided into groups 1 to 4. Group 1 (*C*Las titer value is 0) uninfected citrus leaves were used as a control, group 2 (*C*Las titer value is 1300 ± 500), group 3 (*C*Las titer value is 13,000 ± 2000), group 4 (*C*Las titer value is 32,000 ± 1600). qRT-PCR data were normalized using *Actin* gene. qRT-qPCR was performed in three biological replicates in groups 1, 2, 3, and 4. Bars represent the standard deviation (±SD) calculated for three biological replicates. * represents *p* < 0.05, ** represents *p <* 0.01, *** represents *p* < 0.001 (Student’s *t*-test).

qRT-PCR was carried out for us to further verify the expression patterns of these nine genes, and the data are presented in Figure 5. The primers for qRT-PCR are listed in Table 1. As shown in Figure 5, compared to group 1 (uninfected leaves), five *DnaJ* genes (Ciclev10002746m.1 (*ClDJC47-1*), Ciclev10029600m.1 (*ClDJC24*), Ciclev10011366m.1 (*ClDJB9*), Ciclev10008750m.1 (*ClDJB14*) and Ciclev10030731m.1 (*ClDJC41-1*)) were significantly decreased in group 2 and group 3, while they were increased in group 4. Furthermore, compared to group 1, the other four genes (Ciclev10002196m.1 (*ClDJC82*), Ciclev10016190m.1 (*ClDJC76-1*), Ciclev10001008m.1 (*ClDJC20*) and Ciclev10015385m.1 (*ClDJA1-3*)) were increased in group 2, whereas they were decreased in group 3. However, expression of the *ClDJC24* revealed identical patterns based on qRT-PCR data and the RNA-Seq. In conclusion, the qRT-PCR data showed that expression patterns of most genes were consistent between RNA-seq and qRT-PCR assays (Figure 5), which warranted the reliability of the RNA-seq data. Therefore, the *ClDJC24* gene would be used as a candidate gene for subsequent functional validation experiments.

### 2.7. Synthesis and Characterization of Gold Nanoparticles–Polyethylenimine (AuNPs-PEI)

**Figure 6:**
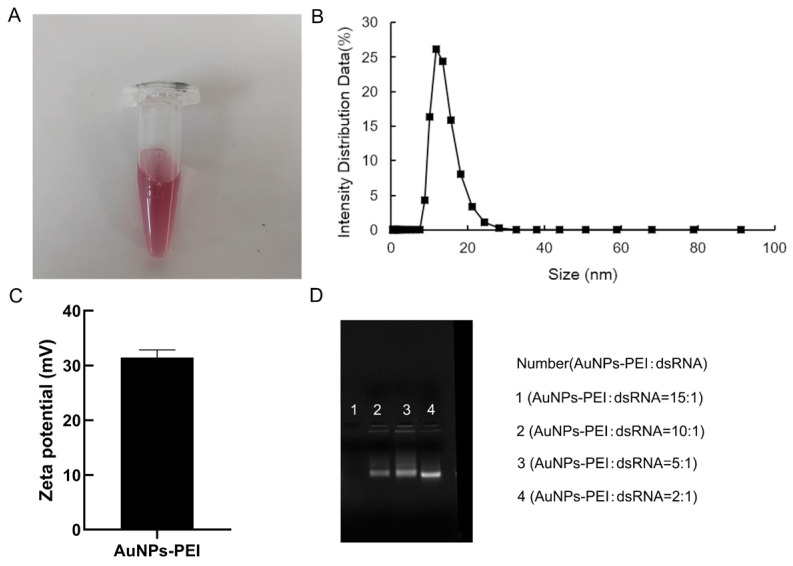
AuNPs-PEI synthesis and characterization. (**A**) The color of AuNPs-PEI solution. (**B**) The particle size of AuNPs-PEI solution and (**C**) Zeta potential of AuNPs-PEI solution. (**D**) Agarose gel electrophoresis of the mixture of AuNPs-PEI solution and dsRNA. A total volume of 16 µL of AuNPs-PEI solution and dsRNA (1 µL) was incubated in different volume ratios (15:1, 10:1, 5:1, 2:1) for 30 min at 28 °C. The mixture was run on 1.2% agarose gel electrophoresis. All experiments were performed with more than three biological replicates.

First, 1 mM gold nanoparticles–polyethylenimine (AuNPs-PEI) was synthesized as previously described [31,32]. In this subject, the AuNPs-PEI adopted the PEI reduction method [32,33,34]. The theoretical size range for nanoparticles that can enter cells is 6–200 nm [35]. As illustrated in Figure 6A, the prepared gold glue solution exhibited a ruby-red color. The results of particle size measurement showed that the particle size of AuNPs-PEI is mainly distributed in the 15–18 nm range (Figure 6B). In Figure 6C, the zeta potential of AuNPs-PEI was 33 mV. Furthermore, the binding assay of AuNPs-PEI and dsRNA was performed. Second, AuNPs-PEI and dsRNA in different volume ratios (15:1, 10:1, 5:1, 2:1) were incubated for 30 min at 25 °C, 50 rpm. After incubation, the binding samples were electrophoresed with 1.2% agarose. As shown in Figure 6D, the result showed that the best bind of AuNPs-PEI and dsRNA increases with decreasing AuNPs volume. According to the result, the 10:1 ratio was chosen for further analysis (Figure 6D). These results indicate that AuNPs-PEI and dsRNA maintain good dispersibility and can easily enter cells.

### 2.8. ClDJC24-dsRNA Silencing Affects the Expression Patterns of Immune Genes in Response to Citrus HLB

**Figure 7:**
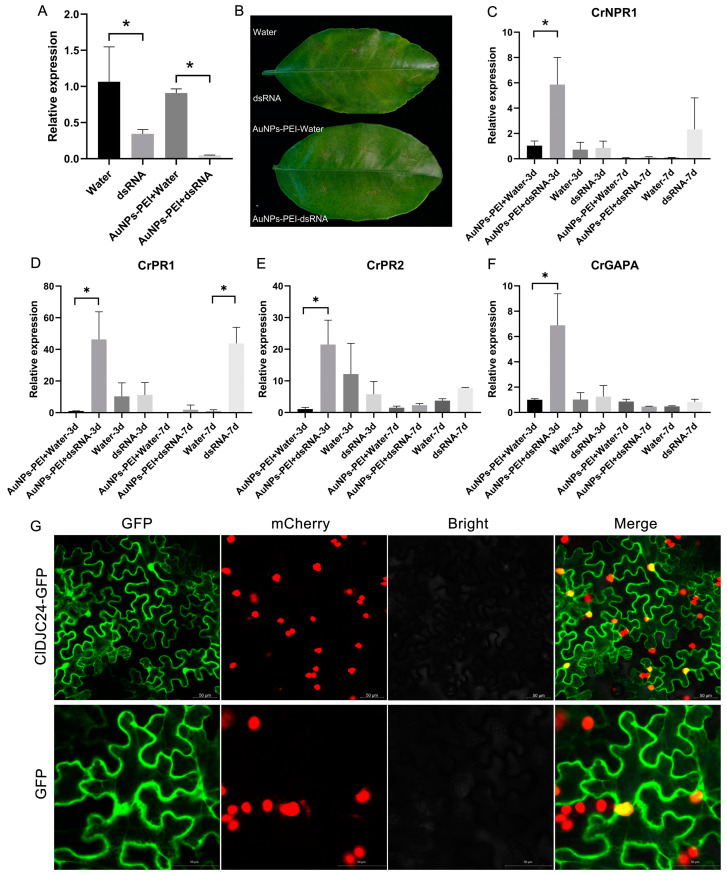
ClDJC24-dsRNA silencing affects the expression patterns of immune genes in response to Citrus HLB. (**A**) The expression level of *ClDJC24* in HLB-infected citrus leaves in 3 d treated with ClDJC24-dsRNA and AuNPs-PEI + ClDJC24-dsRNA, water, and AuNPs-PEI + water was analyzed by qRT-PCR. (**B**) The disease symptoms of dsRNA and AuNPs-PEI + dsRNA, water, and AuNPs-PEI + water treated HLB-infected citrus leaves. (**C**–**F**) represented the expression patterns of immune genes (*CrNPR1*, *CrPR1*, *CrPR2*, *CrGAPA*) in response to Citrus HLB. Data are presented as means ± SDs (*n* = 3). “*” symbol above the columns indicates a significant difference at *p* < 0.05. (**G**) Subcellular localization of *ClDJC24* uses a nuclear-localized marker transgenic *Nicotiana benthamiana* (NLS-mCherry-OE). The GFP empty vector was used as a control. Photographs were taken at 3 d post-infiltration. Scale bar = 50 μm.

To investigate the biological function of *ClDJC24* in response to citrus HLB, wounded HLB-infected citrus leaves were treated with ClDJC24-dsRNA and AuNPs-PEI + CLDJC24-dsRNA, water and AuNPs-PEI + water as controls [36]. As illustrated in Figure 7A, ClDJC24 expression was reduced following treatment with ClDJC24-dsRNA compared to water-treated control leaves, indicating successful silencing of *ClDJC24*. However, compared to the control leaves treated with AuNPs-PEI + water, the expression of dsRNA was significantly decreased following treatment with AuNPs-PEI + CLDJC24-dsRNA (Figure 7A). We measured the *C*Las titers of HLB-infected Citrus leaves treated with ClDJC24-dsRNA and AuNPs-PEI + ClDJC24-dsRNA in 3 d and 7 d. The results showed that the Ct values of *C*Las were not significantly changed in 3 d and 7 d relative to controls. Furthermore, the expression patterns of immune genes *CrNPR1*, *CrPR1,* and *CrPR2* were analyzed by qRT-PCR in response to Citrus HLB (Figure 7C–E). The results showed that *CrNPR1* expression was upregulated in HLB-infected citrus leaves treated with AuNPs-PEI + ClDJC24-dsRNA when compared to AuNPs-PEI + water in 3 d (Figure 6C). The expression of *CrPR1* was increased in the leaves treated with both AuNPs-PEI + ClDJC24-dsRNA and ClDJC24-dsRNA as compared to controls in 7 d (Figure 6D). However, *CrPR2* was significantly expressed in 3 d. In addition, *CrGAPA*, a secretory protein in citrus HLB, was increased in HLB-infected Citrus leaves treated with AuNPs-PEI + ClDJC24-dsRNA in 3 d relative to AuNPs-PEI + water (Figure 7F). The results showed that *ClDJC24* can inhibit immune gene expression of *CrNPR1*, *CrPR1*, *CrPR1,* and *CrGAPA* in 3 d.

To determine the subcellular localization of *ClDJC24*, we designed a ClDJC24-GFP construct using a pUBQ10-GFP vector and transiently expressed it using a nuclear-localized marker transgenic *Nicotiana benthamiana* (NLS-mCherry-OE). As illustrated in Figure 7G, confocal fluorescence imaging of ClDJC24-GFP in NLS-mCherry-OE leaves cells demonstrated the nuclear and cytoplasm localization of ClDJC24-GFP and co-localizes with a nuclear marker. Additionally, ClDJC24-GFP is distributed throughout the entire cell, including the cytoplasm, similar to the localization of the empty vector control. This observation pertains to NLS-mCherry-OE leaves harvested three days post-infiltration.

## 3. Discussion

Huanglongbing (HLB), caused by the bacterium *Candidatus* Liberibacter asiaticus (*C*Las), represents one of the most devastating diseases affecting citrus crops worldwide [27,28]. Despite extensive research, no effective strategies have been identified for curing citrus HLB, and no susceptibility genes associated with HLB have been discovered in citrus species. This absence complicates the application of advanced gene-editing technologies, such as CRISPR-Cas9, to enhance citrus resistance to HLB [37,38,39]. The DnaJ protein family is known to play crucial roles in regulating various physiological processes and is implicated in diverse pathological pathways [40]. However, there is limited research on the mechanisms of interaction between the DnaJ family and plant pathogenic bacteria in citrus species. In this study, we conducted a comprehensive analysis of DnaJ family members in citrus, including their gene structures, conserved domains, phylogenetic relationships, chromosomal locations, and expression patterns in response to HLB infection. We also explored the effects of ClDJC24-dsRNA silencing on the expression patterns of immune-related genes in response to citrus HLB. Previous studies have shown that 117, 115, and 88 *DnaJ* homologs were identified in Arabidopsis [15], rice [16], and wheat [6], respectively. We identified 86 DnaJ family members throughout the Citrus genome. In this study, we identified 86 DnaJ family members across the citrus genome. The lower number of DnaJ members in citrus, compared to Arabidopsis (117) and rice (115), suggests gene loss during evolutionary processes and genome ploidy differences among these species. The isoelectric point (pI) of DnaJs protein sequences ranged from 4.64 (Ciclev10016883m.1) to 10.07 (Ciclev10003089m.1) (Table 1), with 62% of the family members exhibiting a pI greater than 7, a trend consistent with findings in *Capsicum annuum L.* and *Tolypocladium guangdongense* [41,42]. This pI distribution might indicate functional diversification of DnaJ proteins under varying physiological conditions and stress responses in citrus.

Additionally, our analysis revealed an uneven chromosomal distribution of the 86 *DnaJ* genes within the citrus genome. Notably, chromosomes 2 and 8 each contained three tandem duplication events, indicating that the evolution of DnaJ genes has been significantly influenced by gene duplication processes [43,44]. Such duplication events are known to contribute to genetic diversity and functional innovation within gene families. Furthermore, in this study, the analysis of gene structures showed that the number and distribution of introns and exons were highly conservative, and most *DnaJ* gene members contain 4–6 introns. The intron–exon structure offers significant insights into the systematic and comprehensive understanding of the conservation and evolutionary characteristics of gene families [45]. It serves as an important clue for evolutionary studies and functional analyses. This structurally similar evolutionary branch indicates that these genes may corroborate this finding of physiological functions [46].

In this study, we conducted an analysis of the cis-acting elements within the promoters of DnaJ genes in citrus species (Table S2). The results revealed that the *ClDJC24* gene in citrus possesses a significant number of cis-regulatory elements associated with MeJA responsiveness elements (TGACG-motif, TGACG and CGTCA-motif, CGTCA), abscisic acid responsiveness (ABRE, ACGTG) and defense and stress responsiveness elements (TC-rich repeats, GTTTTCTTAC). These findings suggest a potential role for the ClDJC24 gene in regulating various biological activities and responses to environmental stressors. The promoter is a crucial element of genes that regulates the initiation timing and expression levels of gene transcription. The analysis of promoters constitutes the foundation and prerequisite for research on gene expression regulation [47].

These findings suggest that DnaJ genes may play a significant role in regulating various biological activities within citrus plants. Furthermore, based on previously obtained transcriptome data related to Huanglongbing (HLB) from our laboratory [30], we identified nine candidate DnaJ genes for further investigation. Notably, the expression patterns of the DnaJ genes Ciclev10002746m.1 (ClDJC47-1), Ciclev10029600m.1 (ClDJC24), Ciclev10011366m.1 (ClDJB9), Ciclev10008750m.1 (ClDJB14), and Ciclev10030731m.1 (ClDJC41-1) exhibited a consistent decrease in expression levels as the severity of HLB infection escalated, as evidenced by quantitative real-time PCR (qRT-PCR) analysis. This downregulation of DnaJ gene expression in response to HLB infection highlights their potential involvement in the plant’s defense mechanisms against this devastating disease. The correlation between the severity of infection and the expression of these genes underscores the importance of investigating their functional roles in the context of citrus immunity. Future research should focus on elucidating the specific contributions of these DnaJ genes to the overall stress response and exploring their potential applications in enhancing disease resistance in citrus through targeted gene-editing approaches.

To investigate the biological function of the ClDJC24 gene, we examined its interactions with other HSP70 proteins and evaluated the effects of gene silencing in citrus during HLB infection using yeast two-hybrid technology and AuNPs-PEI + dsRNA delivery systems. Our yeast two-hybrid assay results indicated that ClDJC24 does not interact with HSP70 proteins (Figure S3). As a member of the HSP40 protein subfamily, DnaJ genes are characterized by the presence of a conserved DnaJ domain, which typically facilitates interaction with HSP70 proteins [3,7,48,49]. However, our results are inconsistent with previous research findings, suggesting that certain HSP70 family members may have evolved distinct functions in citrus. Additionally, we also synthesized 1 mM gold nanoparticles polyethylenimine (AuNPs-PEI) as previously described [31,32]. The binding assay of AuNPs-PEI and dsRNA revealed that a 10:1 volume ratio effectively silences the target gene (Figure 6D). Previous studies from our laboratory demonstrated that gene silencing was effective at a 9:1 ratio [31,32]. Consistent with these findings, we opted to use the 10:1 volume ratio for further analyses.

In examining the effects of ClDJC24 silencing in HLB-infected citrus leaves, we observed that the cycle threshold (Ct) values of *C*Las did not significantly change in 3, 7, and 14 d post-treatment compared to the controls. This lack of change in *C*Las levels suggests that ClDJC24 may not directly influence *C*Las proliferation under the conditions tested. However, we did find that the expression patterns of immune-related genes *CrNPR1*, *CrPR1*, and *CrGAPA* were inhibited by *ClDJC24* silencing, indicating a potential role for *ClDJC24* in negatively regulating disease resistance. This aligns with previous findings that *SDE420* can inhibit host immunity by interacting with cytoplasmic glyceraldehyde-3-phosphate dehydrogenase (GAPDH), which in turn regulates autophagy [50]. Additionally, *AtGAPA* has been identified as a defense-related target protein of SDEs that inhibits host defenses in *Arabidopsis thaliana* [51]. In addition, subcellular localization of *DnaJ* genes in Citrus was predicted in chloroplast (chlo), nucleus (nucl), and cytoplasm (cyto). Subcellular localization results indicated that the *ClDJC24* was expressed in the nucleus nuclear and cytoplasm (Figure 7G). This result is consistent with the localization of the homologous *DnaJ* gene in *Arabidopsis thaliana* [52]. This nuclear localization suggests that ClDJC24 may play a significant role in the regulation of gene expression and cellular responses to stress, further emphasizing the need for additional studies to elucidate its precise functions in the context of HLB and citrus immunity.

In conclusion, we conducted a comprehensive genome-wide identification and characterization of the DnaJ family genes in Citrus. This study encompassed the analysis of phylogenetic relationships, gene structures, chromosomal locations, and expression patterns for a total of 86 *DnaJ* genes. Based on the results of the expression analysis of *ClDJC24*, the functional role of *ClDJC24* was further investigated. *ClDJC24* gene may be a negative regulator of disease resistance in Citrus for its silencing effect. Yeast two-hybrid (Y2H) assay results demonstrated that the ClDJC24 protein does not interact with HSP70 proteins. This study offers valuable insights for further functional characterization and establishes a theoretical basis for the development of disease-resistant Citrus cultivars.

## 4. Materials and Methods

### 4.1. Identification of DnaJ Genes in Citrus

A Hidden Markov Model (HMM) profile of the DnaJ domain (Pfam: PF00226) was downloaded from the Pfam database (http://pfam.xfam.org, accessed 11 April 2024) and was adopted for the identification of *DnaJ* genes in the Citrus genome (https://www.citrusgenomedb.org/, accessed 11 April 2024). The threshold was 0.01, and the parameter was set to the default value. To confirm the presence of the DnaJ domain, a batch search of the sequences of all the obtained *DnaJ* genes in the Citrus was conducted using the online databases SMART (http://smart.embl.de/smart/, accessed 15 April 2024), NCBI Conserved Domain Search databases (NCBI CDD) (https://www.ncbi.nlm.nih.gov/cdd/, accessed 15 April 2024) and Pfam (http://pfam.xfam.org/, accessed 15 April 2024). Redundant sequences were manually deleted. Furthermore, the amino acid numbers (AAs), molecular weights (Mw), and isoelectric points (pI) of the identified *DnaJ* genes were obtained using the ExPasy website (http://web.expasy.org/protparam/, accessed 5 May 2024). Additionally, using the online WoLF PSORT (https://www.genscript.com/wolf-psort.html, accessed 8 June 2024) predicted subcellular localization.

### 4.2. Chromosomal Locations and Gene Structure Analysis and Cis-Element Analysis of DnaJ Genes in Citrus

According to the locations of the identified *DnaJ* genes in Citrus in the database, they were obtained from annotated gff3 files. All the *DnaJ* genes were mapped to the banana chromosomes with MapChart software according to their positions in the database [53]. An online program of the gene structure display server (GSDS2.0) (https://gsds.gao-lab.org/index.php, accessed 10 June 2024) was applied to draw the exon/intron organization of each *DnaJ* gene in Citrus by comparing the cDNAs with their corresponding full-length sequences [54]. The MEME tool (http://meme-suite.org/tools/meme, accessed 15 June 2024) was used to identify conserved motifs of 86 *DnaJ* gene proteins [55]. Parameters applied were as follows: the number of motifs searched was set as 10, and the limits of motif widths were between 6 and 50 residues [56]. For cis-acting element analysis, genomic DNA sequences in the upstream 1500 bp region of all *DnaJ* genes were obtained, and the sequences were identified using the PlantCARE database (http://bioinformatics.psb.ugent.be/webtools/plantcare/html/, accessed 5 October 2024) [57].

### 4.3. Phylogenetic Analysis and Heat Map Construction of DnaJ Genes from Citrus

The phylogenetic analysis of the DnaJ gene family in Citrus was performed using MEGA X with 1000 bootstrap replicates. The phylogenetic tree was constructed employing the neighbor-joining (NJ) method and pairwise gap deletion mode [56]. The domain information was retrieved from the SMART database (http://smart.emblheidelberg.de/, accessed 2 August 2024). To further insights into the expression patterns of *DnaJ* genes, FPKM counts were regarded as thresholds to define significant differences in gene expression. *DnaJ* gene expression levels were analyzed in relation to the expression patterns in HLB. RNA-Seq data were visualized with heat maps using TBtools [58]. In the transcriptome data, sample A (A-1, A-2, A-3): post-treatment sample (new tissues), 90 days after FOS and CAC combination therapy; Sample B (B-1, B-2, B-3): pre-treatment sample. A-1, A-2, A-3, and B-1, B-2, B-3 represented no HLB-infected and HLB-infected with three biological replicates, respectively. The treatment method for Group A is as follows: the roots of the citrus trees were first soaked in 1 L of Fosthiazate solution at a concentration of 480 µg/mL, applied as a soil drench (75% Fosthiazate from Hebei Sannong Agricultural Chemical Co., Ltd., Shijiazhuang, China). After 3 to 5 days, we applied 1 L of 200 µg/mL CAC to each citrus sample in the greenhouse through root drenches (the Fosthiazate or CAC dosage was increased to 3 L per tree in the orchard). The samples were collected 90 days post-treatment.

### 4.4. RNA Extraction and Quantitative Reverse Transcription qRT-PCR

Total RNA was extracted with the CTAB method [51,59]. Briefly, qRT-PCR was performed on a StepOne real-time PCR system with ChamQ Universal SYBR qPCR Master Mix (Vazyme Biotech, Nanjing, China) following the manufacturer’s instructions. The total RNA was treated to eliminate any potential contamination with DNA using gDNA Eraser (TaKaRa, Dalian, Japan). Subsequently, DNA-free total RNA was reverse transcribed into cDNA with a reverse transcription kit (TaKaRa, Dalian, Japan), following the manufacturer’s instructions. Real-time Quantitative Polymerase Chain Reaction (qRT-PCR) was performed as described by Li et al. [56]. Primer pairs of 9 *DnaJ* genes were designed using the online software Primer 3 (http://primer3.ut.ee/, accessed 8 April 2024). The primers are listed in Table 2. Gene relative expression levels were normalized to that of the reference gene Citrus *Actin* [60]. The expression levels were calculated using the 2^−ΔΔCt^ method [61]. There were three independent biological replicates of each sample type, and the data were shown as the mean ± standard deviation.

### 4.5. Subcellular Localization

*ClDJC24* genes were cloned from cDNA generated from citrus leaves following the manufacturer’s instructions (Max Master Mix, Vazyme Biotech, Nanjing, China). The amplified fragments were ligated into the pUBQ10-GFP empty vector using the In-Fusion Cloning Kit (Vazyme Biotech, Nanjing, China). The primers used for plasmid construction are listed in Table S1. Successfully constructed ClDJC24-GFP vectors were transformed into Agrobacterium tumefaciens strain GV3101. Agrobacterium-mediated transient expression in *Nicotiana benthamiana* leaves for 72 h was carried out as previously described in Huang’s methods [36,62]. Using LSM880 confocal microscope (Carl Zeiss, Oberkochen, Germany) subcellular localization of ClDJC24-GFP was observed. The excitation wavelength for GFP was 488 nm, and the detection wavelength ranged from 500 to 550 nm [51].

### 4.6. Yeast Two-Hybrid (Y2H) Assays

The yeast two-hybrid method was performed according to the protocol described by Huang et al. [36,62]. The Y2H system was used to identify protein–protein interactions in vitro. The coding sequence (CDS) of *ClDJC24* was cloned in frame into the bait vector pGBKT7, while the CDSs of HSP90 and HSP70 were cloned in frame into the prey vector pGADT7, which all used the In-Fusion Cloning Kit (Vazyme Biotech, Nanjing, China). The primers used for plasmid construction are listed in Table S1. Following the manufacturer’s protocol, the Y2H Gold yeast strain was cotransformed with the resulting bait and prey vectors to identify positive interactions (Clontech, Mountain View, CA, USA). The co-transformants pGBKT7-53 + pGADT7-T and pGBKT7-lam + pGADT7-T serve as the positive and negative controls, respectively. The positive interactors were screened on SD/–leucine–tryptophan–adenine–histidine (SD/-LWAH) medium supplemented with X-α-gal at 30 °C in the dark for approximately 3 days. Meanwhile, SD/–leucine–tryptophan (SD/-LW) medium was used to screen the positive transformants. X-α-gal was used to confirm positive interactions by turning the colonies blue. The experiment’s analysis involved observing whether yeast colonies grew on SD/-LWAH and SD/-LW media and whether colonies on SD/-LWAH medium with added X-α-gal turned blue. If the colonies grew on SD/-LW and turned blue on SD/-LWAH after approximately 3 days, the results indicated positive interactions. The plates were then photographed using a Nikon camera.

### 4.7. AuNPs-PEI Synthesis

Prepare 14 mL PEI (branched polyethylenimine, 25KD, Shyuanye, Shanghai, China) solution (final concentration 0.2 mM) and 1 mL chloroauric acid (HAuCl_4_, Aladdin, Shanghai, China) solution (final concentration 2 mM) with double distilled water (ddH_2_O), respectively. Added PEI solution to HAuCl_4_ solution and slowly heated to 85 °C until the solution was ruby red in color. Continued to stir until the solution cooled to room temperature to obtain AuNPs-PEI [32]. Centrifuged at 70,403× *g* for 15 min to remove the free PEI from the supernatant and resuspended the pellet in 200 mL ddH_2_O. Particle size and zeta potential detection 800 mL of AuNPs-PEI or AuNPs-PEI-pNPR1-GFP ten-fold dilution were tested by Zetasizer Nano ZS90 (Malvern Instruments, Malvern, PA, USA) [35,36].

### 4.8. Gene Silencing

The dsRNAs, approximately 300 bp in size and complementary to *ClDJC24*, were synthesized using the T7 RNAi Transcription Kit following the manufacturer’s protocol (Vazyme Biotech, Nanjing, China). The PCR technique was utilized to add the T7 promoter sequence to both ends of the RNA interference (RNAi) target fragments. Details of the primers used for PCR are provided in Table 1. The sequences containing the T7 promoters were purified and used as templates for dsRNA amplification. Subsequent infection assays were conducted to establish the effects of knocking out target genes, following the method described by Huang [36,62]. HLB-infected citrus leaves were detached and uniformly wounded using the tip of a pipette head. Subsequently, 10 μL of dsRNA and AuNPs-PEI solution at a concentration of 500 ng/μL were applied to the abaxial surface of the leaves with 0.02% Silwet L-77 (Solarbio Science & Technology, Beijing, China). As a control, corresponding sites on the same leaves were treated with 10 μL of water and AuNPs-PEI solution containing 0.02% Silwet L-77. Silwet L-77, a surfactant, was used to enhance the adsorption and spread of dsRNA or water on the Citrus leaves [36]. Ten HLB-infected Citrus leaves were used for each treatment. Each experiment was independently repeated three times. The leaves were also harvested for qRT-PCR to verify gene silencing in 3 d, 7 d, and 14 d.

### 4.9. Detection of Citrus Huanglongbing (HLB) Bacterial Titer

Using copy number as the indicator: the Ct value is first obtained using plant genomic DNA as the template, and the copy number is then calculated based on the generated standard curve. A higher copy number indicates a higher bacterial titer. Using genomic DNA from Catharanthus roseus infected with Huanglongbing (HLB) as a template, specific primers HLBas and HLBr were used to amplify the 16S rDNA sequence of *C*Las. The 16S rDNA sequence was verified by 1.5% agarose gel electrophoresis, and the product was recovered and ligated into the pMD19-T vector to construct the recombinant plasmid pMD19-T-16S rDNA (p16S rDNA), which was then transformed into *E. coli* DH5α. Single colonies were selected for colony identification, and if correctly identified, the culture was expanded, and plasmids were extracted and quantified. The plasmids were then diluted with DEPC water to 10^−1^, 10^−2^, 10^−3^, 10^−4^, 10^−5^, 10^−6^, 10^−7^, and 10^−8^ gradients.

Using the diluted plasmid p16S rDNA as a template, absolute quantitative PCR was performed with a 2× TaqMan Fast qPCR kit (B639275, Sangon Biotech (Shanghai) Co., Ltd China). The primer and probe sequences are HLBas (TCGAGCGCGTATGCAATACG), HLBr (GCGTTATCCCGTAGAAAAAGGTAG), and HLBp (AGACGGGTGAGTAACGCG). The following program: 95 °C for 5 min; 95 °C for 30 s; 60 °C for 30 s; 40 cycles. Each sample was run with at least three technical replicates, and a standard curve was plotted based on the copy number of *C*Las and Ct values. The formula for calculating the copy number is as follows: A = (M × N) ÷ (L × D). A: CLas copy number; M: mass concentration (g/mL) = DNA (ng/mL) × 10^−6^; N: Avogadro’s constant (6.022 × 10^23^ molecules/mol); L: DNA length, which is the number of base pairs in the recombinant plasmid containing the target gene, in kb; D: Conversion factor (6.6 × 10^5^ g/mol/kb).

### 4.10. Limitations of the Study Related to the Species Source of the Data

The database used for this transcriptome sequencing is *Citrus clementina*, while the expression analysis, including qRT-PCR and the expression levels of immune genes (*CrNPR1*, *CrPR1*, *CrPR2*, *CrGAPA*), is based on *Citrus reticulata*. Since there was no database available for *Citrus reticulata* at the time, we chose a phylogenetically close database for the transcriptome sequencing. In this study, to ensure consistency with the transcriptome database, we selected the *Citrus clementina* database for analysis.

## Figures and Tables

**Table 1:** Physicochemical analysis of *DnaJ* genes in Citrus.

Annotation Number	Gene Name	AAs	Mw (kDa)	pI	Subcellular Localization	Arabidopsis Ortholog	Clade
Ciclev10030731m.1	ClDJC41-1	803	90.54	8.82	nucl	DJC41	I
Ciclev10030729m.1	ClDJC41-2	806	89.42	8.61	nucl	DJC41	I
Ciclev10004367m.1	ClDJC41-3	779	86.57	8.88	nucl	DJC41	I
Ciclev10000081m.1	ClDJC44	1142	127.65	8.78	nucl	DJC44	I
Ciclev10001879m.1	ClDJC45	317	35.15	8.54	cyto	DJC45	I
Ciclev10000551m.1	ClDJC46	646	73.81	9.27	nucl	DJC46	I
Ciclev10004241m.1	ClDJC67	1000	113.3	5.43	nucl	DJC67	I
Ciclev10014483m.1	ClDJC84 ^$^	689	76.14	8.8	nucl	DJC84 ^$^	I
Ciclev10028303m.1	ClDJLD13 ^$^	490	54.74	8.7	nucl	DJLD13 ^$^	I
Ciclev10029411m.1	ClDJLD9 ^$^-1	167	18.84	5.4	cyto	DJLD9 ^$^	I
Ciclev10029403m.1	ClDJLD9 ^$^-2	169	19.19	5.64	cyto	DJLD9 ^$^	I
Ciclev10017157m.1	ClDJLD9 ^$^-3	131	14.9	9.45	nucl	DJLD9 ^$^	I
Ciclev10029096m.1	ClDJLD9 ^$^-4	259	28.71	8.91	nucl	DJLD9 ^$^	I
Ciclev10020330m.1	ClDJA1-1	418	46.94	6.65	nucl	DJA1	II
Ciclev10028531m.1	ClDJA1-2	416	46.16	6.06	nucl	DJA1	II
Ciclev10015385m.1	ClDJA1-3	416	46.46	6.01	nucl	DJA1	II
Ciclev10031530m.1	ClDJA3	448	49.38	9.11	nucl	DJA3	II
Ciclev10008033m.1	ClDJA4	513	56.45	8.9	chlo	DJA4	II
Ciclev10025609m.1	ClDJA6	443	47.83	9.41	chlo	DJA6	II
Ciclev10025441m.1	ClDJA7	499	53.56	8.89	chlo	DJA7	II
Ciclev10020947m.1	ClDJA8	345	39.08	5.93	cyto	DJA8	II
Ciclev10016063m.1	ClDJB1-1	305	34.34	8.91	cyto	DJB1	II
Ciclev10012139m.1	ClDJB1-2	339	37.39	9.31	cyto	DJB1	II
Ciclev10015887m.1	ClDJB1-3	331	37.21	7.61	nucl	DJB1	II
Ciclev10008858m.1	ClDJB10	337	36.91	7.69	nucl	DJB10	II
Ciclev10015820m.1	ClDJB3	344	37.54	8.97	cyto	DJB3	II
Ciclev10032269m.1	ClDJB6	295	32.81	9.22	cyto	DJB6	II
Ciclev10001008m.1	ClDJC20	478	53.19	8.96	chlo	DJC20	II
Ciclev10020899m.1	ClDJC51	350	39.13	8.98	nucl	DJC51	II
Ciclev10003893m.1	ClDJC79	385	43.69	5.73	cyto	DJC79	III
Ciclev10028788m.1	ClDJC80	340	38.25	7.71	cyto	DJC80	III
Ciclev10001330m.1	ClDJC81	410	45.14	5.5	cyto	DJC81	III
Ciclev10026825m.1	ClDJC23	101	11.35	8.64	nucl	DJC23	IV
Ciclev10029600m.1	ClDJC24	119	13.13	9.06	nucl	DJC24	IV
Ciclev10011261m.1	ClDJC2	646	73.62	8.55	nucl	DJC2	IX
Ciclev10008750m.1	ClDJB14	358	40.48	8.75	nucl	DJB14	V
Ciclev10005299m.1	ClDJC35	352	41.26	8.54	nucl	DJC35	V
Ciclev10005578m.1	ClDJC30	282	32.34	8.95	nucl	DJC30	VI
Ciclev10033027m.1	ClDJC33	139	16.3	4.96	nucl	DJC33	VII
Ciclev10002824m.1	ClDJC50	133	16	5.18	nucl	DJC50	VII
Ciclev10016883m.1	ClDJC28	181	21.11	4.64	chlo	DJC28	VIII
Ciclev10026366m.1	ClDJC29	242	26.78	5.04	chlo	DJC29	VIII
Ciclev10002746m.1	ClDJC47-1	159	17.86	4.98	nucl	DJC47	VIII
Ciclev10022709m.1	ClDJC47-2	148	16.62	4.8	chlo	DJC47	VIII
Ciclev10030891m.1	ClDJC17	671	74.96	5.7	plas	DJC17	X
Ciclev10004408m.1	ClDJB16	741	82.34	9.16	chlo	DJB16	XI
Ciclev10019555m.1	ClDJC36/ClDJC37	555	63.4	8.78	nucl	DJC36/DJC37	XI
Ciclev10015402m.1	ClDJB11	414	45.84	6.18	nucl	DJB11	XII
Ciclev10031692m.1	ClDJB12	410	46.33	8.72	nucl	DJB12	XII
Ciclev10011610m.1	ClDJC16	484	54.19	6.66	cyto	DJC16	XIII
Ciclev10014072m.1	ClDJC31	1214	133.52	5.66	chlo	DJC31	XIII
Ciclev10004152m.1	ClDJC62	1408	153.92	6.23	nucl	DJC62	XIII
Ciclev10012465m.1	ClDJC61	270	31.88	8.28	chlo	DJC61	XIV
Ciclev10011366m.1	ClDJB9	577	63.66	9.36	nucl	DJB9	Others
Ciclev10003422m.1	ClDJC10	182	20.53	4.49	nucl	DJC10	Others
Ciclev10016093m.1	ClDJC19	300	35.17	9.31	chlo	DJC19	Others
Ciclev10026659m.1	ClDJC22	163	18.43	5.62	chlo	DJC22	Others
Ciclev10019624m.1	ClDJC25	540	60.21	8.96	chlo	DJC25	Others
Ciclev10006058m.1	ClDJC26-1	170	19.16	8.74	chlo	DJC26	Others
Ciclev10022177m.1	ClDJC26-2	218	24.76	10.03	nucl	DJC26	Others
Ciclev10007224m.1	ClDJC27/ClDJC74	2592	283.9	5.91	nucl	DJC27/DJC74	Others
Ciclev10029128m.1	ClDJC32	250	29.6	9.21	nucl	DJC32	Others
Ciclev10030221m.1	ClDJC34	412	45.96	9.3	nucl	DJC34	Others
Ciclev10000724m.1	ClDJC57	567	64.79	8.7	nucl	DJC57	Others
Ciclev10002163m.1	ClDJC65	268	31.78	9.71	nucl	DJC65	Others
Ciclev10003690m.1	ClDJC66-1	127	14.44	8.92	nucl	DJC66	Others
Ciclev10009810m.1	ClDJC66-2	147	16.06	9.34	chlo	DJC66	Others
Ciclev10002683m.1	ClDJC66-3	170	18.9	9.8	chlo	DJC66	Others
Ciclev10003089m.1	ClDJC66-4	147	16.62	10.07	chlo	DJC66	Others
Ciclev10010382m.1	ClDJC66-5	104	12	6.27	nucl	DJC66	Others
Ciclev10006484m.1	ClDJC66-6	166	18.42	6.18	cyto	DJC66	Others
Ciclev10021147m.1	ClDJC69	325	36.21	10.05	chlo	DJC69	Others
Ciclev10012897m.1	ClDJC70	181	20.51	9.54	nucl	DJC70	Others
Ciclev10027520m.1	ClDJC71	528	60.83	8.22	nucl	DJC71	Others
Ciclev10026217m.1	ClDJC72	285	32.93	9.56	nucl	DJC72	Others
Ciclev10028974m.1	ClDJC73	289	33.14	5.27	nucl	DJC73	Others
Ciclev10002209m.1	ClDJC75	259	29.66	6.02	chlo	DJC75	Others
Ciclev10016190m.1	ClDJC76-1	281	31.72	9.24	chlo	DJC76	Others
Ciclev10031498m.1	ClDJC76-2	456	50.3	9.02	chlo	DJC76	Others
Ciclev10017707m.1	ClDJC77	340	38.33	6.27	chlo	DJC77	Others
Ciclev10002309m.1	ClDJC78	241	28.82	9.55	nucl	DJC78	Others
Ciclev10002196m.1	ClDJC82	262	29.42	7.01	chlo	DJC82	Others
Ciclev10006857m.1	ClDJC83 ^$^	287	33.73	9.57	nucl	DJC83 ^$^	Others
Ciclev10019373m.1	ClDJC9	604	69.09	5.49	nucl	DJC9	Others
Ciclev10009451m.1	ClDJLD4	215	24.23	5.85	chlo	DJLD4	Others
Ciclev10019092m.1	ClDJLD8	703	79.48	5.42	chlo	DJLD8	Others

Note: ^$^ newly found J-proteins in *Arabidopsis* according to the study by Zhang et al.

**Table 2:** The primer sequences used for RT-PCR.

Genes	Forward Primers	Reverse Primers
Ciclev10002196m.1	TATTCATGTGTAAAAACAGCCCCTA	GTGCGTCCAAGATACTCTCAAGTAA
Ciclev10002746m.1	ATGAGAACAAGAGGTCAATGTACGA	TATTCATCATGGAGACCATCTCTTG
Ciclev10029600m.1	GGAAATGTCGGCAAATGAGTTTATC	TTCTGAGGAAAGCGAAGAAGAAGA
Ciclev10011366m.1	TTCAAGTTCATTTGGTTTTGGTCTA	TAAATTTATGGCTCTGATGTTTCCA
Ciclev10008750m.1	AAGAGGGGTGAATTTTTATGTGAAG	AATGGGGAGTCTCCTTTATAAAACC
Ciclev10030731m.1	GAGTACCTCCGAACTTATCTCAACA	AGTGTATGTGTTTCCTGTCATGGTA
Ciclev10016190m.1	AAGAAATCAAAGAGGCTTACAGGAA	TGGAAGCATCATAATCTTTTCTGAG
Ciclev10001008m.1	ACATTGCCTCAATCGATACTTCATA	ATACACCTCCTAGCCAAAAACATTC
Ciclev10015385m.1	CATAAATGATGAAGGAATGCCTATG	CTTCACACTCATCCAGTTCCATATC
ClDJC24-dsRNA	CGGCTACAACAGCTTGTTCA	GCCGTTGAGTCCTTGTCTTC
CrNPR1	TGAGGGATTATGAAGTTGGGTTTG	CTTGTCCAGAATGTCTAGGAGGTG
CrPR1	ACAGTGAGACAATTGCATGGAG	AGCACAACCTAAACGCACAG
CrPR2	CTCTACGCTCCGAATCAAGCAACT	CGGCGATGTAGCGAAACTTG
CrGAPA	TACACTGGCGACCAAAGGCTACTC	GCGGCGTTCACCTCTTCTGC
Actin	TCTGTACTCAACCGTCTTCC	ATCACAACCACTCTTCATCTC

## Data Availability

Data contained within the article.

## Supplementary Materials

The following supporting information can be downloaded at: https://www.mdpi.com/article/10.3390/ijms252211967/s1.
