# Correction: Tian et al. Genome-Wide Identification of the DnaJ Gene Family in Citrus and Functional Characterization of *ClDJC24* in Response to Citrus Huanglongbing. *Int. J. Mol. Sci.* 2024, *25*, 11967

**DOI:** 10.3390/ijms26010411

**Published:** 2025-01-06

**Authors:** Yuzhen Tian, Xizi Wang, Huoqing Huang, Xin Deng, Baihong Zhang, Yixuan Meng, Libo Wu, Hang Chen, Yun Zhong, Wenli Chen

**Affiliations:** 1 MOE Key Laboratory of Laser Life Science & Institute of Laser Life Science, Guangdong Provincial Key Laboratory of Laser Life Science, Guangzhou Key Laboratory of Spectral Analysis and Functional Probes, College of Biophotonics, School of Optoelectronic Science and Engineering, South China Normal University, Guangzhou 510631, China; tianyzh@126.com (Y.T.); wangxizi1103@163.com (X.W.); 2021023392@m.scnu.com (Y.M.); 2023023373@m.scnu.edu.cn (L.W.); ccc2020xxsc@126.com (H.C.); 2 Institute of Fruit Tree Research, Guangdong Academy of Agricultural Sciences, Key Laboratory of South Sub-Tropical Fruit Biology and Genetic Research Utilization, Ministry of Agriculture and Rural Affairs, Guangdong Provincial Key Laboratory of Science and Technology Research on Fruit Tree, Guangzhou 510640, China; hqhuang07@163.com; 3 Department of Biomedical Sciences, City University of Hong Kong, Kowloon Tong, Hong Kong SAR, China; xindeng@cityu.edu.hk; 4 Shenzhen Research Institute, City University of Hong Kong, Shenzhen 518057, China; 5 Institute of Nanfan & Seed Industry, Guangdong Academy of Science, Guangzhou 510640, China; zhangbyhome@126.com

In the original publication [1], there was an error in Figure 7G as published. The incorrect image was uploaded due to a review oversight. The corrected Figure 7G appears below. The authors state that the scientific conclusions remain unaffected. This correction has been approved by the Academic Editor. The original publication has also been updated.

## Figures and Tables

**Figure 7:**
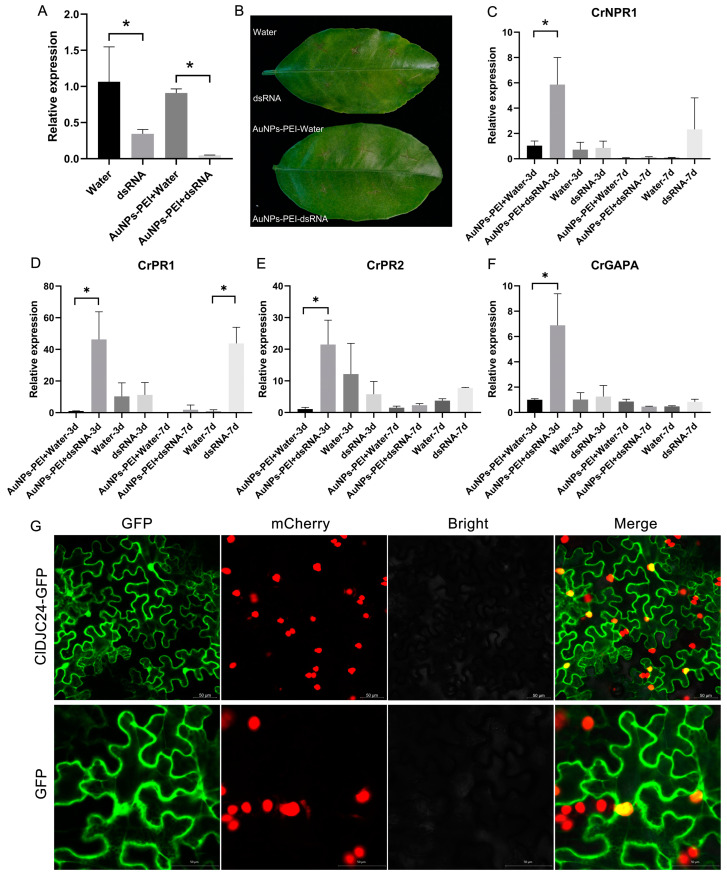
ClDJC24-dsRNA silencing affects the expression patterns of immune genes in response to Citrus HLB. (**A**) The expression level of *ClDJC24* in HLB-infected citrus leaves in 3 d treated with ClDJC24-dsRNA and AuNPs-PEI + ClDJC24-dsRNA, water, and AuNPs-PEI + water was analyzed by qRT-PCR. (**B**) The disease symptoms of dsRNA and AuNPs-PEI + dsRNA, water, and AuNPs-PEI + water treated HLB-infected citrus leaves. (**C**–**F**) represented the expression patterns of immune genes (*CrNPR1*, *CrPR1*, *CrPR2*, *CrGAPA*) in response to Citrus HLB. Data are presented as means ± SDs (*n* = 3). “*” symbol above the columns indicates a significant difference at *p* < 0.05. (**G**) Subcellular localization of *ClDJC24* uses a nuclear-localized marker transgenic *Nicotiana benthamiana* (NLS-mCherry-OE). The GFP empty vector was used as a control. Photographs were taken at 3 d post-infiltration. Scale bar = 50 μm.
