# Supplementary material for: Genome-Wide Identification of the DnaJ Gene Family in Citrus and Functional Characterization of ClDJC24 in Response to Citrus Huanglongbing
Source: Int J Mol Sci. 2024 Nov 7;25(22):11967. doi: 10.3390/ijms252211967 (PMC11593701; doi:10.3390/ijms252211967)
Supplement: Supplementary file 1 [file ijms-25-11967-s001.zip › Supplementary.pdf]

Table S1. The primer sequences used for RT-PCR

Genes	Forward Primers	Reverse Primers
DnaJ-3-GFP	CGCACTAGTGCGCCCATGGCAGCAGCG GCTTCT	CCCGGTACCCGCCCCCCAGCACTGA TCGGTCTCCCA
DnaJ-3-BD	GCCGAATTCCCGGGGATCATGGCAGCA GCGGCTTCT	CGCTGCAGGTCGACGGATCCAGCAC TGATCGGTCTC
Hsp90-1-AD	GGTGGGCATCGATACGGGATCATGGCA TCAGAGACAGAGACGTTT	TGCAGCTCGAGCTCGATGGATATCA ACTTCCTCCATCTTGCTGCC
Hsp70-2-AD	GGTGGGCATCGATACGGGATCATGAAG ATCAAGAACAAAGCATTAGCT	TGCAGCTCGAGCTCGATGGATTTTCA TTAGCTCATAAACCTCTTTGATCAC
Hsp70-3-AD	GGTGGGCATCGATACGGGATCATGGCT GGAAAAGGCGAAGGT	TGCAGCTCGAGCTCGATGGATATCC ACCTCCTCAATCTTGGG
Hsp70-4-AD	GGTGGGCATCGATACGGGATCATGGAC GGGAAAGAAGAGGTACTAGCA	TGCAGCTCGAGCTCGATGGATGTTC ATGACCAGACCCAAGATTAGCGA

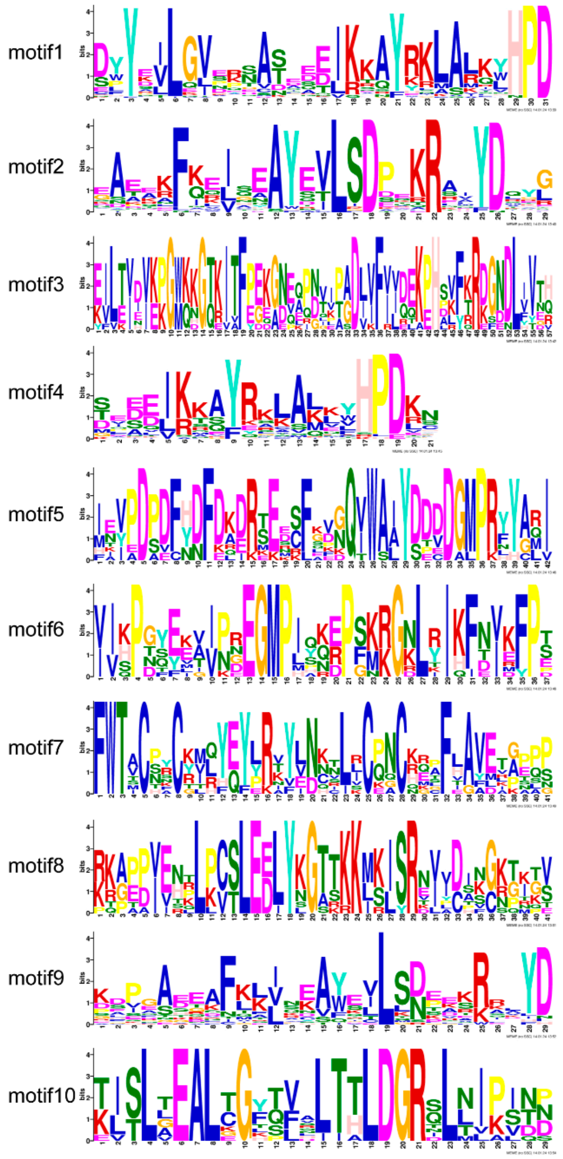

Figure S1 The detailed information for each motif

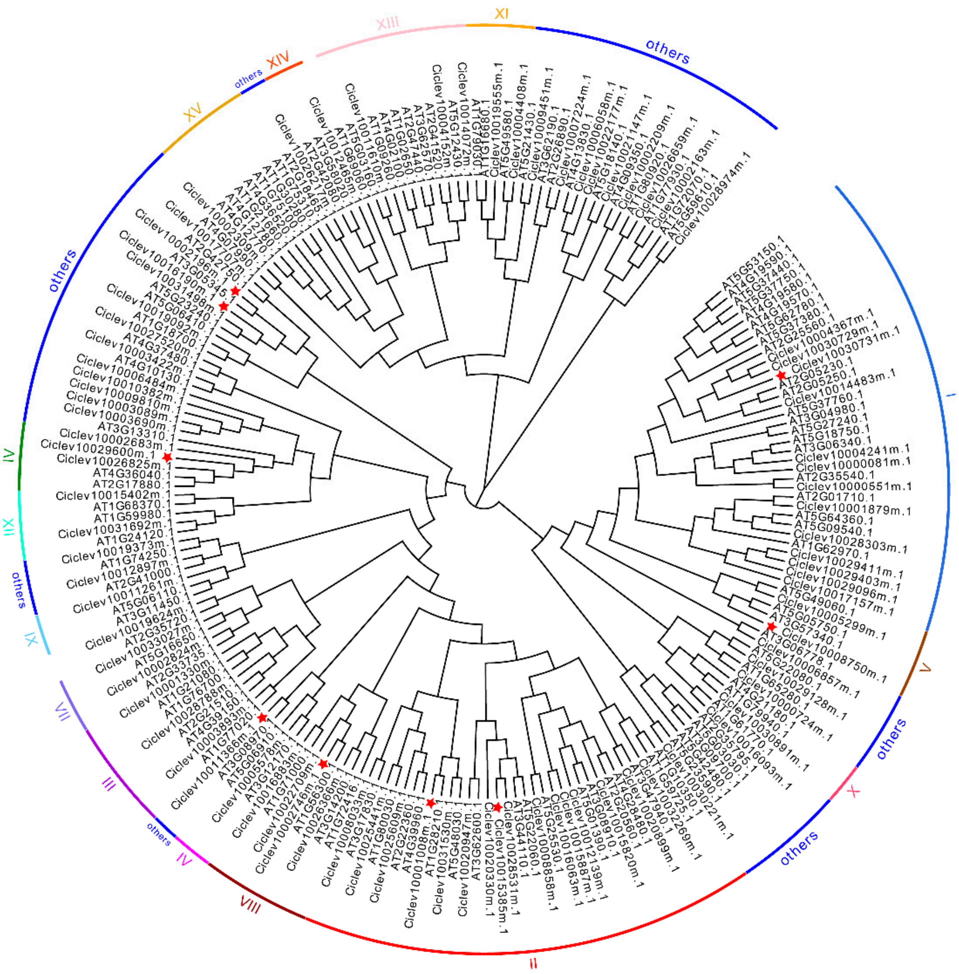

Figure S2 Phylogenetic analysis of DnaJ proteins from Arabidopsis and Citrus. ClustalW was applied for the alignment of protein sequences. Neighbor-joining method with 1000 bootstrap replicates was utilized to construct the phylogenetic tree in MEG7.0 software. Each DnaJ clade is indicated in a specific color. Asterisks indicate the nine genes identified based on transcriptome data.

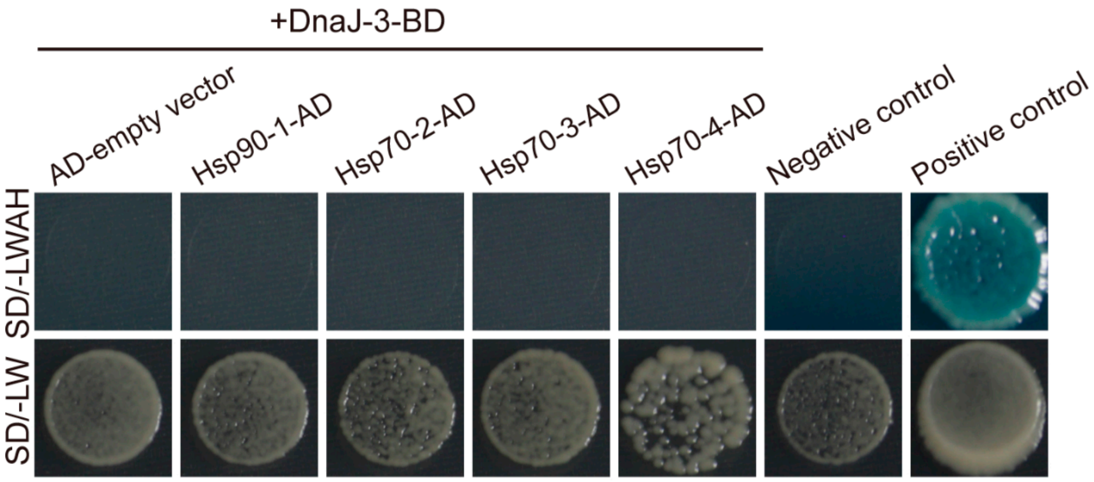

Figure S3 DnaJ-3 interacts with Hsp90 and Hsp70 proteins. The cotransformants of p53+pGADT7-T and lam+pGADT7-T are presented as positive control and negative control, respectively. SD/-LWAH is presented as SD/-leucine-tryptophan-adenine-histidine, SD/-LW is presented as SD/-leucine-tryptophan.
